# The Chemical Scaffold of Theranostic Radiopharmaceuticals: Radionuclide, Bifunctional Chelator, and Pharmacokinetics Modifying Linker

**DOI:** 10.3390/molecules27103062

**Published:** 2022-05-10

**Authors:** Holis Abdul Holik, Faisal Maulana Ibrahim, Angela Alysia Elaine, Bernap Dwi Putra, Arifudin Achmad, Achmad Hussein Sundawa Kartamihardja

**Affiliations:** 1 Department of Pharmaceutical Analysis and Medicinal Chemistry, Faculty of Pharmacy, Universitas Padjadjaran, Sumedang 45363, Indonesia; faisal17011@mail.unpad.ac.id (F.M.I.); angela19001@mail.unpad.ac.id (A.A.E.); bernap19001@mail.unpad.ac.id (B.D.P.); 2 Department of Nuclear Medicine and Molecular Theranostics, Faculty of Medicine, Universitas Padjadjaran/Hasan Sadikin General Hospital, Bandung 40161, Indonesia; a.achmad@unpad.ac.id (A.A.); hussein2017@unpad.ac.id (A.H.S.K.); 3 Oncology and Stem Cell Working Group, Faculty of Medicine, Universitas Padjadjaran, Bandung 40161, Indonesia

**Keywords:** radiopharmaceuticals, theranostics, radionuclide, chelator, pharmacokinetic-modifying linker

## Abstract

Therapeutic radiopharmaceuticals have been researched extensively in the last decade as a result of the growing research interest in personalized medicine to improve diagnostic accuracy and intensify intensive therapy while limiting side effects. Radiometal-based drugs are of substantial interest because of their greater versatility for clinical translation compared to non-metal radionuclides. This paper comprehensively discusses various components commonly used as chemical scaffolds to build radiopharmaceutical agents, i.e., radionuclides, pharmacokinetic-modifying linkers, and chelators, whose characteristics are explained and can be used as a guide for the researcher.

## 1. Introduction

Theranostics is a term in the medical field to define the combination of therapeutic and diagnostic techniques by a suitable pharmaceutical agent [1]. It is an effort to improve therapeutic interventions after imaging is performed to find the target entity of the disease [2]. The term theranostic has long been known since the combination of therapy and diagnostics first appeared about 70 years ago, but, nowadays, theranostic is developing rapidly [2]. One of the developments of theranostic techniques is through the utilization of radionuclides in the chemical scaffold of pharmaceuticals. This developed agent is then further called “theranostic radiopharmaceutical, also called radiotheranostics.” Radiotheranostics is one of the most advanced applications of the theranostic field; therefore, diagnosis and therapeutic intervention are closely related in radiotheranotics. An essential aspect of radiotheranostics is that the patient’s molecular target for both diagnostics and therapy is precisely the same [2]. Therefore, radiotheranotics offers a breakthrough method compared to traditional diagnostic and therapeutic approaches, which commonly address different entities/molecules in treating a single disease. As expected, radiotheranostics development has attracted many in medicinal chemistry, molecular biology, and clinical medicine in recent years.

In radiotheranostics, a pharmaceutical agent (drug) is needed to be a carrier molecule that introduces the radionuclide to its target. Radionuclides are then used as a source of radiation in radiotheranostics that are responsible for diagnosing or treating various diseases. Although several important nonmetallic (organic) radionuclides (^18^F, ^11^C, ^13^N, ^15^O, ^124^I, etc.) have been around for quite some time, metallic radionuclides are of particular interest for radiopharmaceutical development because of their more expansive range of nuclear properties (half-life, decay characteristics, etc.), rich coordination chemistry, and availability in the nature. In nuclear medicine, radiopharmaceuticals are mainly used in diagnostic radio imaging and internal radiotherapy [2].

**Figure 1:**
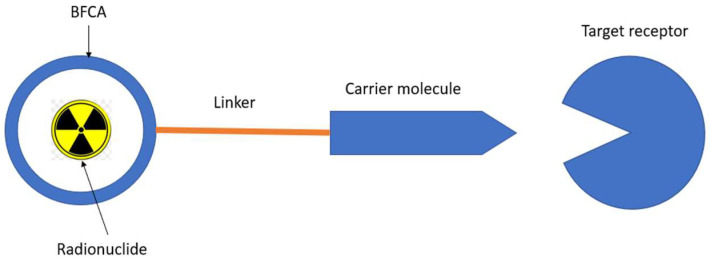
Ligand–BFCAs–radioisotope as a commonly designed radiopharmaceutical.

The ligand–BFCAs–radioisotope design is the most common radiotheranostics structure (Figure 1) [3]. The carrier molecule acts as a vehicle of radiopharmaceuticals by bringing the radionuclide to the molecule of interest, enriching the therapeutic radioisotope locally in or around the tumor. A peptide, antibody, amino acid, or small molecule is often used as a carrier molecule. Chelators (Bifunctional Chelating Agents/BFCAs) build a chelate complex between the carrier molecule and the radioisotope [2]. BFCAs have been influential in improving the clinical use of radiopharmaceutical peptides and protein bioconjugates in nuclear medicine [4]. In between BFCA and carrier molecule, the pharmacokinetics modifying linkers (PKMs) often used as a bridge which also useful for modifying pharmacokinetic profile of the whole complex and creating a certain space between carrier and radionuclide-chelator complex.

The utilization of a single organic molecule that incorporates either a therapeutic or a diagnostic radionuclide is the disctinctive feature of radiotheranostics. It is assumed that both will have almost identical pharmacokinetics. Therefore, quantitative analysis of positron emission tomography (PET) or single-photon emission computed tomography (SPECT) images enables accurate estimation of the absorbed radiation dose in both the target lesion (therapeutic effects) and the non-target tissues (side effects). The major challenges in developing a radiotheranostics system are (i) identifying and (ii) optimizing an appropriate combination of diagnostic and therapeutic radionuclides and matching chelators for the selected radionuclides.

In this review, the explanation of radionuclides, chelators, and PKMs that are commonly used to build radiopharmaceutical scaffolds has been explained as a guide for the researcher to build new agents of radiopharmaceuticals.

## 2. Radionuclides

Radionuclides are isotopes that emit radiation or have excess nuclear energy, making them chemically unstable and tend to change into another atom. Various types of radiation can be emitted by radionuclides e.g. alpha particles, beta particles, and gamma energy (Table 1).

**Figure 2:**
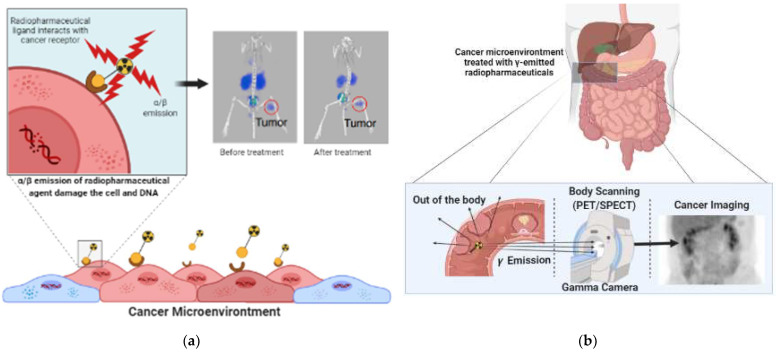
The illustration of radiopharmaceutical use for cancer therapy and diagnosis: (**a**) The utilization of alpha- and beta-emitted radiopharmaceuticals for cancer therapy [8]. (**b**) The utilization of gamma-emitted radiopharmaceuticals for cancer diagnosis (imaging).

Figure 2 illustrates how a specific radiopharmaceutical binds into a target molecule in a specific cell and damages its DNA (Figure 2a) or emit gamma ray to produce a clear cancer images (Figure 2b). In order to deliver the highest possible total radiation dose to malignancies, therapeutic radiopharmaceuticals should be able to be absorbed in large quantities and retained sufficiently long enough at the target. Therefore, long-lived radionuclides are the best fit. A pure imaging radioparmaceutical, on the other hand, should feature a quick absorption in the target and rapid clearance from the body due to their short-time requirement for observation in the clinic as well as avoiding non-specific binding in the body [8].

**Figure 3:**
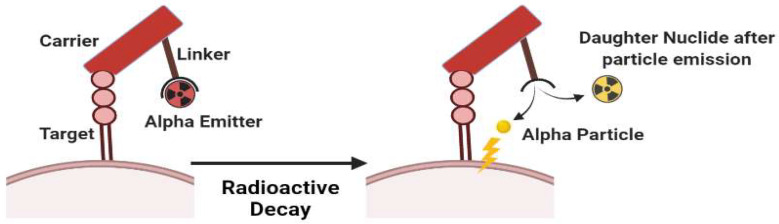
Scheme of complex instability of alpha emitter due to recoil energy effect.

Therapeutic radiopharmaceuticals are commonly labeled with α or β^−^ emitters. Alpha emitters (α) follow a reduction in the number of atomic nuclei in the form of helium particles (^4^α_2_). Unfortunately, the recoil energy effect experienced by the daughter’s nuclide during alpha emission may causes radiotoxicity. Figure 3 simply shows that bond breakage between the alpha emitter and its chelator will always occur after emission, which might cause significant injury due to the distribution of free-form daughter nuclide in the body. The distribution of recoiling daughters is influenced by three mechanisms: (1) distance traveled owing to recoil energy, (2) diffusion processes, and (3) active transport such as blood transportation [9,10].

Another type of emission for therapeutic purposes is high-energy (β^−^) particles that delivered into targets using peptides (e.g., ^90^Y-DOTATOC) or antibodies (e.g., ibritumomab tiuxetan [Zevalin]) [11]. The β^−^ emitter has such a "long” range of radiation in tissue (1–12 mm) due to its low mass, which may affect normal cells surrounding the tumor microenvironment by breaking membrane thickness and DNA denaturation [12].

For imaging purposes, radionuclides release photons that interact minimally with intervening tissue, either as rays (e.g., ^99^m-Tc) or as annihilation photons (e.g., ^68^Ga) generated by positron (β^+^) decay (Figure 2b) [13].

### 2.1. Radionuclides for Diagnosis Purposes

SPECT and PET have become increasingly popular cancer imaging techniques. The radiopharmaceuticals use a gamma (γ) or beta (β^+^) emitted radionuclide to selectively interact with a target tissue. In PET and SPECT tracers, a variety of radionuclides have been utilized, including isotopes ranging from ^11^C (t_1/2_= 20 min) to ^124^I (t_1/2_= 4.2 days), as shown in Table 2 [14].

In the PET scan, the annihilation of positron caused by the collision between positron and electron in the body generates gamma rays in two opposite directions which can be detected by the PET scanner to build a radiogram. The acceptable resolution of radiopharmaceuticals should be about ~5 mm. The position of annihilation (collision) varies between the different radionuclides, this is the reason behind the various resolution of the radiogram for each radiopharmaceutical. For example, a positron released by ^18^F (E+ = 250 keV) travels 1 mm before being annihilated, but a positron emitted by ^68^Ga (E+ = 830 keV) travels 35 mm before being annihilated. As a result, PET pictures acquired with ^68^Ga have poorer resolution than those produced with ^18^F [15,16].

On the other hand, SPECT uses the gamma rays produced by a radioactive isotope to be further screened by the device. Because gamma rays are solitary events, unlike annihilation photons, they must be detected by putting a lead collimator between the source and the detector which then provides information about each source of radiation in the cell of interest [17]. Any photons that interact with the detector must have come from a parrarel source to the detector’s face. The energy of the photon being photographed is an essential factor to consider. Low-energy photons (~70 keV) are strongly attenuated by tissue, resulting in distortions in the pictures. If the photon energy is too great, the demand for similarly thick collimators becomes prohibitive. Photon energies of the order of 140 keV are predicted from these two variables, indicating that ^99m^Tc is widely used for SPECT diagnosis [18].

**Table 2:** Radionuclides for diagnosis purposes.

Radionuclide	Half-Life	Mode of Decay	Energy (KeV) (%Abundance)	Indication (in Radiopharmaceutical Form)	References
^99m^Tc	6.02 h	γ	140.5 (89%)	(*l,l*-[^99m^Tc]Tc-ECD) Functional imaging of the brain *, [99m Tc-MDP] bone scintigraphy *	[19,20,21]
^111^In	67.3 h	EC	171 (90%) 295 (94%)	(^111^In-pentetreotide) imaging of neuroendocrine tumors *, (Capromab Pendetide) for metastatic prostate cancer *, and leukocyte marking for invitro purposes *	[22,23,24,25,26]
^18^F	109.7 min	β^+^ EC	635 (97%) 1655 (EC) 3%	FDGPET radionuclide for cancer * and Piflufolastat PET radionuclide for protate cancer imaging *	[27,28]
^11^C	20.4 min	β^+^	960 (100%)	Imaging of tyrosine kinase receptor *****, [^11^C]Flumazenil for GABA **** imaging, [^11^C]mZIENT for imaging serotonin receptor *****, and 11C-coenzyme Q10 myocardial imaging *****	[29,30]
^133^Xe	5.27 days	γ	81 (38%)	Cerebral blood flow, Xe Technegas for lung perfusion imaging **	[31,32]
^201^Tl	73 h	γ	135 and 167	imaging of soft tissue and bone tumors, detection of recurrence in gliomas	[33]
^51^Cr	27.7 days	γ	320 (9.8%)	Red blood cell labeling, 51-EDTA for GFR measurement ***	[34,35]
^67^Ga	78.3 h	EC γ	EC (100%) γ (93 (39%), 300 (17%), and 185 (21%))	Imaging skeletal infection, ^67^Ga–Citrate for CSF flow imaging ****	[36,37,38]
^68^Ga	68 min	β^+^	890 (90%)	Diagnosis or imaging of myocardial perfusion use Ga-68 Galmydar ****, pulmonary perfusion ****, and PSMA for prostate cancer *.	[39,40]
^123^I	13 h	EC	159	Ioflupane I-123 Injection * Injection Dopamine transporter for parkinson’s diagnosis	[41,42]
^125^I	59.4–60.2 d	EC	28.5	Evaluation of glomerular filtration rate and imaging of thyroid, and 125 Iodine Seeds for brachytherapy in solid tumor *.	[43,44,45,46,47]
^82^Rb	75 s	β^+^	776	^82^Rb(Rb)^+^**** for myocardial ischemia and brain tumors imaging.	[48,49]
^13^N	9.97 min	β^+^	492 (100%)	^13^N-ammonia * for myocardial perfusion and blood flow imaging in tissue.	[50]
^166^Ho	26.8 h	β^−^ γ	1.774 (50%) 80.57 (6.6%)	^166^Ho-chitosan ***** for diagnosis of liver cancer	[51,52]
^89^Zr	78.4 h	β^+^	395 (23%)	Diagnosis of various types of tumor and cancer (pancreatic, lymphoma, liver, colorectal, and prostate) (^89^Zr-trastuzumab, ^89^Zr-J951, ^89^Zr-lumretuzumab) *****	[53]
^61^Cu	3.3 h	β^+^ EC γ	1220, 1150 (62%); 940, 560 (38%); 380 γ (3%)	^61^Cu-ATSM ***** imaging of tumor hypoxia.	[54]
^64^Cu	12.7 h	β^+^ β^−^ γ	657 (19%), 141 (38%) 511 (43%),	^64^Cu-SAR-bisPSMA *** Imaging for prostate, ^64^Cu-DOTA-Trastuzumab *** breast cancer, ^64^Cu-ATSM *** diagnosis of cervical cancer, ^64^Cu-DOTA-Daratumumab **** multiple myeloma, and ^64^Cu-Cl_2_ urological malignancy.	[55,56]

Note: * FDA approved. ** Clinical trial phase III. *** Clinical trial phase II. **** Clinical trial Phase I. ***** Pre-clinical Studies.

a. ^99m^-Technetium

^99m^Tc (t_1/2_ = 6,02 h, E = 140.5 keV (89%)) is widely used for SPECT, with ^99m^Tc radiopharmaceuticals accounting for more than 85% of all nuclear medicine studies [57]. The emission and half time of ^99m^Tc are almost ideal for convenient preparation of radiopharmaceuticals and imaging applications [58]. Several in vitro studies regarding the toxicity of ^99m^Tc have been performed. The results of studies showed that [^99m^Tc]TcO_4_^−^ was able to induce DNA damage in breast cancer epithelial cells and reduce cell survival rate when the [^99^m-Tc]TcO_4_^−^ was transported into cells. About 30 mBq/cell of cellular concentration of ^99^m-Tc was required to reduce the survival rate to 37%. Currently, ^99m^Tc is widely used as a radiodiagnostic agent because it has several advantages e.g., widely available, can produce a variety of complexes with desired characteristics due to multi-oxidation state, sufficient half-life (6.02 h) for the preparation of ^99m^Tc radiopharmaceuticals (in hospitals or centralized radiopharmacies), and decays into ^99^Tc which has low toxicity (weak beta emission and very long half-life) [58,59,60].

^99m^Tc has traditionally been generated via a ^99^Mo/ ^99m^Tc generator using parent ^99^Mo created by the fission of highly enriched ^235^U. The ^99^Mo is separated from the ^99^MoO_4_^2−^ and immobilized through the alumina column [59]. By eluting the generator with 0.9% (isotonic) saline solution, ^99m^Tc is produced as ^99m^Tc O_4_^−^. Schaffer et al. demonstrated that a biomedical cyclotron could create 7.7 GBq (208 mCi) of ^99m^Tc after 1.5 h of irradiation with an 18 MeV proton beam.

The primary distinctions between rhenium and technetium are their redox behaviors and kinetics. They are found in oxidation levels ranging from +7 to +1 and belong to the same group of transition metals (VIIB) as manganese. Tc(V) and Re(V) produce structurally similar complexes, but the formation circumstances and stability of the resultant products differ, with ^188^Re complexes being easier to oxidize [60].

A reducing agent, most often SnCl_2_, chelating (e.g., DTPA, DOTA, and SAR), and a buffer are usually included in a ^99m^Tc kit. The ^99m^Tc O_4_^−^ solution in saline is simply injected into the vial to make the ^99m^Tc complex [61,62]. Tc(V)-oxo or -dioxo (d^2^) complexes are formed by reducing ^99m^Tc O_4_^−^ with SnCl_2_, yielding square-pyramidal or octahedral complexes, respectively, with tetradentate having higher in vivo stability and simpler chemical modification, and so becoming the popular choice. Amido thioether thiol (AATT) and single amino acid chelate (SAAC) systems are examples of this tetradentate kind of complex [63].

b. ^111^Indium

^111^In (t_1/2_ 2.8 d) is the most typical application for cell labeling, which has the advantage of being compatible with SPECT rather than with PET. For the tagging of a wide range of cell types, two compounds have proven especially useful: [^111^In]In(oxinate)_3_ and [^111^In]In(tropolonate)_3_ with In(oxinate)_3_ have been the most used in clinic [14]. ^111^In is a source of gamma radiation used for diagnosis, is also a source of low-energy auger electrons, and has a short distribution range. The presence of electrons outside the cell can be neglected, but when these electrons are inside the cell or around the cell nucleus, they can have a highly toxic effect on the cell’s DNA. A comparative study of dosimetric estimation of ^111^In showed that ^111^In was transported into cancer cells in a cumulative concentration range of about 3.7–9.4 mGy/MBq, whereas in the liver ^111^In had a cumulative concentration range of 0.6–0.4 mGy/MBq. The result of the study indicated that ^111^In accumulates higher in cancer cells when compared to normal cells and can induce a cytotoxic effect against cancer cells [23].

Proton irradiation of enriched ^112^Cd targets with the ^112^Cd(p,2n)^111^In reaction is the most frequent method for producing ^111^In. At proton energy of approximately 25 MeV, this reaction can be performed in intermediate energy cyclotrons [63]. Indium, like gallium, has just one stable oxidation state in water: +3. However, due to its much greater size at 62–92 pm for ionic radius (4–8 Å), In(III) achieves coordination numbers of seven and even eight in its complexes. Indium complexes formed by the acyclic chelators EDTA and DTPA are highly thermodynamically stable. The hexadentate chelator in the seven-coordinate In-EDTA structure approximates a pentagonal bipyramidal geometry [64].

c. ^67^Galium, and ^68^Gallium

Gallium has a +3 oxidation state in an aqueous solution, which is comparable to ^111^In. Ga^3+^ is a hard metal (small and highly charged cation) with an ionic radius of 4–6 CN (0.4–0.6 Å) that prefers to bind chelator with numerous anionic oxygen and/or nitrogen donor sites (e.g., DOTA, NOTA, and HBED) [65,66]. The variation of Ga^3+^ coordination number from 3 to 6 support octahedral complexes, the most common and the most stable Ga3+-based radiopharmaceuticals. pH sensitivity is a significant problem in Ga^3+^ labeling; gallium quickly hydrolyzes at pH>3, converting it to insoluble Ga(OH)_3_. As a result, it is commonly labeled in acidic circumstances (pH 3–4), with the use of an intermediary ligand as an option (e.g., citrate or oxalate) [18,67].

Two radionuclides, ^67^Ga and ^68^Ga, have dominated the development of gallium-based radiopharmaceuticals. The low (γ) energy emitter ^67^Ga (t_1/2_ = 78.2 h) decays solely via electron capture (EC) [68,69]. Gallium-67 also generates high-energy (6.3 KeV) and long-range Auger electrons, which has spurred interest in ^67^Ga for treatment purposes [70]. Gallium-67 is commonly made by bombarding a ^nat^Zn or isotopically enriched ^68^Zn target with the nuclear reactions ^68^Zn(p,2n)^67^Ga (photon energy range between 15 and 30 MeV) and/or ^67^Zn (p,n)^67^Ga (photon energy range between 10 and 20 MeV) and/or ^67^Zn (p,n)^67^Ga (photon energy range between 10 and 20 MeV [71].

^68^Ga (t_1/2_ = 67.7 min) on the other hand, is a (β^+^) emitter (89%) with a mean (β^+^) energy of 0.830 MeV, allowing it to be utilized for diagnostic imaging with PET. Gallium-68 is generated by the ^nat^Ga (p,xn)^68^Ge nuclear reaction, which is then absorbed on a column containing either an inorganic (e.g., TiO_2_, Al_2_O_3_, and SnO_2_) or organic (polymeric) stationary phase, and then eluted as ^68^GaCl_3_ with 0.1–1 M HCl for further radiolabeling [72,73]. 

^68^Ga and ^111^In are used for inflammatory diagnosis and tumor imaging. Gallium(III) (Ga^3+^) has comparable properties to Fe-III when in the body to bind to transferrin and lactoferrin and be transported to sites of inflammation. Compared with In(III), Gallium has advantages over indium in imaging osteomyelitis (bone infection) and chronic inflammation due to its ability to bind neutrophil cell membranes. In another utilization, ^111^In is most suitable with peptides labeled for the somatostatin receptor and antibody receptor for prostate tumor imaging [74,75]. Several recent studies of cytotoxicity of ^67^Ga have shown that ^67^Ga caused DNA damage higher than ^111^In per Bq concentration. The level of cellular radioactivity required by ^67^Ga to kill 50–90% of breast cancer cells is 1.5–6 times less than ^111^In. In addition, ^67^Ga also has a lower level of DNA damage than ^111^In if the radionuclide was separated from DNA, which caused ^67^Ga to have a smaller DNA damage effect on non-targeted cells [38].

d. ^61^Copper and ^64^Copper

^61^Cu is produced from the cyclotron (^61^Ni(p,n)^61^Cu) whereas ^64^Cu is produced from ^64^Ni(d,2n)^64^Cu with γ emissions (43%) and produced from ^64^Ni(p,n)^64^Cu with β^+^ emissions (19%). The half-life of ^61^Cu is shorter than ^64^Cu, making ^64^Cu the most used for radiodiagnostic development due to its higher stability [76]. The coordination chemistry of copper, which is quite diverse ~4 to 6, makes Cu isotopes stably functional with BFCs. On the other hand, Cu has two states of oxidation, Cu(I) and Cu(II); thus, the complex remains stable and soluble with chelators phosphine-P and thioether-S (weak donor chelator). Because of the d9 configuration, Cu(II) complexes are more kinetically able to dissociate to ligands than Cu(I) with Cu(I) d10 configurations. Therefore, the compatible chelator Cu radionuclides are generally macrocyclic chelators, making Cu(II) complexes stably conjugated with the ligands [77,78]. The cytotoxicity study of ^64^Cu radionuclide conjugated with carriers and chelators showed that the accumulated concentration of ^64^Cu was 3.1–6.0 times higher in cancer cell models than in normal cell models. These results indicate higher efficiency of ^64^Cu in cancer cells. The result shows that ^64^Cu has the potential to be used as a radionuclide for cancer therapy or diagnosis [76].

e. ^89^Zirconium

The half-life of Zr-89 (t_1/2_ = 78.4 h) is the most suitable for antibody-based radiopharmaceuticals, due to their slow pharmacokinetics. It has relatively low positron energy (395 keV), which results in ideal radionuclides for PET with high-resolution imaging and is also more stable and safer in vivo. Hence, ^89^Zr has more uses than ^124^I-based agents for clinical applications. In addition, ^124^I produces different energies, 723 keV (10.4%), 1691 keV (10.9%), and 603 keV (63.0%), which may lead random result to imaging. However, proper handling during high abundance production is necessary to lower the risk of high energy and penetrating photons (909 keV) [53].

f. ^18^Fluorine

Despite the benefits of ^68^Ga, such as its ability to be obtained from a generator or its metallic nature of molecules via coordination chemistry, ^18^F continues to have a privileged position among the radionuclides used in PET imaging. The sensitivity and spatial resolution of ^68^Ga PET imaging are lower than those of ^18^F PET. With a bond strength of around 670 kJ/mol, aluminum forms more stable complexes with fluorine than with other halogens. Furthermore, because the Al-F bond is extremely stable in vivo, tiny quantities of the aluminum fluoride complex are compatible with organisms. With a maximum coordination number of six, the Al^3+^ ion can be complexed by an appropriate chelator and form a ternary complex (fluorine aluminum–chelator) in the presence of fluoride ions. If the ligand’s valency permits it, it prefers to assume an octahedral geometry [79,80].

The relatively fast in vivo pharmacokinetics of peptide bioconjugates are consistent with the fluorine-18 half-life, making them ideal for [^18^F]AlF radiolabeling. Good labeling yields of up to 74% have also been produced employing a NODA-MPAA-conjugate (IMP485), a pentavalent chelator better suited to [^18^F]AlF, according to McBride, W.J et al. [81].

### 2.2. Radionuclides for Therapy Purposes

High-energy, short-range radiotherapy is thought to be adequately targeted the tumor tissue without inflicting considerable harm to normal tissue. Radiotherapy should have a high tumor-to-background ratio, be selective in its penetration, and be eliminated quickly by the kidneys. Radionuclides that primarily emit (β^−^) particles, alpha particles, and/or Auger electrons have been used in medicinal radiopharmaceuticals thus far (Table 3) [82]. Auger electrons (mostly in ^67^Ga) are extremely low-energy electrons (1–10 keV) produced by radionuclides decaying via electron capture. These particles have a high Linear energy transfer (LET) (4–26 keV/m) and a tissue range of less than a single cell diameter (1–20 m), making them ideal for nucleus targeting [83].

LET is a popular method for predicting the possible harm that a nuclide might produce in a biological system. Particles having a high LET generate ionizing radiation that quickly disperses in tissue. Particles having a low LET, on the other hand, attenuate their energy slowly, allowing them to deposit energy over a wider range of tissue.

a. ^186^Rhenium and ^188^Rhenium

^188^Re has a half-life of about 17 h and emits β^–^ particles (2120.4 keV, 71.1%; and 1965.4 keV, 25.6%), while ^186^Re has a longer t_1/2_ (90 h) and emits (β^–^) particles (1077 keV, 71%; and 939 keV, 22%) resulting in a longer tissue penetration (10–11 mm). Based on physical properties, ^188^Re isotopes are excellent for radiotherapy of malignant tumors [91]. Some of the chemical properties of ^186^Re and ^186^Re are quite similar to ^99^m-Tc because of their periodic linkage, but ^186^Re and ^188^Re have a lower redox state than ^99^m-Tc, making them incompatible with several BFCA of ^99^m-Tc. Ram et al. find the BFCA for peptide-based antibody which has a more stable complex for ^99^m-Tc and ^186^Re [92]. 

Based on phase II clinical trials and dosimetry tests of radiopharmaceuticals labeled with ^188^Re, ^188^Re-HEDP has effective pain relief in patients with breast or prostate cancer bone metastases (80% of 15 patients), lung cancer bone metastases (46% of 27 patients), renal cancer (50% of 61 patients), and liver cancer (55.56% of 64 patients). The dosimetry test of ^188^Re-HEDP showed the maximum tolerated dose was 3.3 GBq, and the radiation-absorbed dose in normal bone marrow was still tolerable and did not cause hematological toxicity [92].

b. ^225^Actinium

Actinium is present naturally in association with uranium. ^225^Ac [t_1/2_= 10 d; α emission 5793 keV (18.1%), 5830 keV (50.7%)] is derived from the decay of ^233^U as well as the transmutation of neutron of ^226^Ra by successive n, γ capture decay reactions via ^227^Ac, ^228^Th, and ^229^Th. In the clinical trials, ^225^Ac can be obtained from U.S. Department of Energy, Oak Ridge National Laboratory (ORNL) in Oak Ridge, TN, United States of America, and the Institute for Transuranium Elements in Karlsruhe, Germany. The ^225^Ac from both places were produced from 233U and the long-term storage was done in ORNL [10]. 

^225^Ac is an α emitter that has a recoil event (see Figure 3) as a challenge when it is complexed with a certain BFCA. There are broad techniques offered to deal with those problems; the first method is to use a nano-carrier capacity to hold the recoiling offspring, such as zeolites or liposomes. Piotrowska et al., 2013, utilized zeolites as transporters for ^224^Ra and found that under circulating blood conditions, the fraction of recoiled daughters (^212^Bi, ^212^Pb, and ^208^Tl) escaped from the zeolites is minimal [93]. The second strategy is to guarantee that the radiopharmaceutical is quickly absorbed by tumor cells and that any residual unabsorbed material is quickly eliminated from the body. Antibodies have received a lot more interest in the field of alpha radionuclide treatment. The third strategy is to place or inject alpha-emitting radionuclides directly into/near the tumor tissue, as Cordier et al. did in Phase I clinical trials using a radiopharmaceutical coded as “^213^Bi-DOTA-substance P” which was locally injected in gliomas [94].

c. ^90^Yttrium

^90^Y is in equilibrium with its parent isotope ^90^Sr and then decays to form the stable ^90^Z. ^90^Y is formed from ^89^Y being bombarded with neutrons in a nuclear reactor. ^90^Y has a half-life of about 2.67 days and emits a large amount of β^−^ (2.27 MeV) and a little β^+^ and γ emission, capable to penetrate tissues up to 11 mm. Due to the high energy β^−^ emission by ^90^Y, the beta particle radiation not only reaches the target but also rapidly reaches the surroundings of the target cell. About 90% of the radiation is absorbed in a path length of 5 mm (about 100–200 cells). The ^90^Y emitted beta particles can, directly and indirectly, disrupt cell integrity. Directly, beta particles will damage the DNA structure so that it cannot be repaired. Meanwhile, the emitted beta particles can increase the amount of toxic free radicals in the cytosol indirectly (known as a secondary radiation effect) [95,96].

^90^Y is a good example of both medicinal and diagnostic isotopes being accessible in the same element. It has a lengthy half-life of 2.7 days, which is long enough to achieve radiotherapy’s critical dosage levels. High energy causes the cell to penetrate deeply, which is ideal for solid tumors. Because of its larger tissue penetration range, ^90^Y was predicted to have a bigger influence on tumor reduction [95].

The strong affinity of unchelated yttrium for bone and liver dominates the bioinorganic chemistry of Y^3+^, which necessitates the employment of macrocycle chelators and emphasizes the importance of complex stability. Yttrium(III) is much bigger, having an ionic radius of 6–9 Å, giving it the ability to achieve coordination numbers of 7 and 10 in its complexes. The octadentate lanthanide chelator DOTA offers a nearly perfect match with associated high affinity due to the higher coordination number requirement of Y(III) [96,97].

Although (n,γ) reactions on yttrium metal or yttrium oxide can generate yttrium-90, the resultant product has a poor specific activity. It may also be made in a nuclear reactor by the ^90^Zr(n,p)^90^Y reaction. After irradiation, the Zr starting material is removed with HNO_3_ and mandelic acid, yielding a solution comprising the ^90^Y daughter and the ^90^Sr parent, which may be removed from the ^90^Y product by retaining it on a DOWEX cation column [97].

d. ^177^Lutetium

In the application of radionuclide-based therapy, ^177^Lu is now becoming the market leader. ^177^Lu (t_1/2_ = 6.7 d) is a β^−^ (0.497 MeV), γ (113 keV, 6%) and (208 keV, 10%) emitters. The development of ^177^Lu to be a theranostic agent is very prospective, its utilities are not only a post-treatment scans be acquired, but patient dosimetry can also be performed [98,99]

In 2018, [^177^Lu]Lu-DOTATATE (Lutathera) was approved by the FDA for use as a cancer treatment following a phase III clinical trial. Studies regarding PRRT combining [^177^Lu]Lu-DOTATATE with [^90^Y]Y-DOTATATE have shown that kidney injury and myelosuppression are rare side effects. Hematotoxicity due to PRRT can occur due to irradiation and destruction of hematopoietic cells. The acceptable dose of PRRT for bone marrow is 2 Gy [100].

There are two methods of ^117^Lu production, direct and indirect methods. The direct approach uses the ^176^Lu(n,γ) ^177^Lu nuclear reaction to irradiate highly enriched ^176^Lu targets with neutrons. In the reaction ^176^Yb(n, γ) ^177^Yb → ^177^Lu, the indirect approach employs highly enriched ^176^Yb as a target material and needs chemical separation of ^177^Lu from excess Yb [101]. Polycarboxylate ligands (DOTA, NOTA, NODAGA, DTPA, and DOTRP) have been demonstrated to be the most successful choice for developing a BFC capable of binding ^177^Lu and producing a radioconjugate with adequate stability in an aqueous solution and under biological conditions for ^177^Lu labeling. The chemical bonds produced by the Lu^3+^ ion have a strong ionic nature, requiring negatively charged hard donor elements such as oxygen for stable coordination. Negative oxygen atoms in polycarboxylate ligands appear to have a function in providing a strong ionic connection with the ionic metallic core. This is an important element in lowering the enthalpy of thermodynamically favorable processes [102].

e. ^153^Samarium

^153^Sm has physical properties being beta emission minus 0.71 MeV (50%) and 0.81 MeV (20%) and an emitted gamma emission 103.2 keV (28%). Beta particles from ^153^Sm can penetrate soft tissue up to a maximum distance of 3 mm and can penetrate bone up to 1.7 mm, so it is used to reduce bone pain and bone loss due to cancer-causing illnesses and is also used for gamma imaging biodistribution [87,88].

## 3. Bifunctional Chelator Used in Radiopharmaceutical Agents

A bifunctional chelating agent (BFCA) is a component of radiopharmaceuticals as a complexor (chelating agent) in metal-based radiolabeling to build a stable link radiometal to carrier molecules. The use of BFCA needs to consider several aspects e.g. the stability of the organo-metallic complex as well as the radiopharmaceutical interaction against drug target in the presence of chelate [103]. BFCA is covalently bound to the carrier molecule and sometimes bound or linked by a pharmacokinetic-modifying linker (PMK) in between (Figure 1). 

The selection of BFCA is determined by its physicochemical properties e.g. oxidation state, and coordination number of radiometal. Each radiometal has its specific characteristic that requires suitability against its BFCA according to the electron donor-acceptor number of the atoms. Ideally, selected BFCA can form stable chelates or complexes with high thermodynamic stability and kinetic inertness results [104]. In addition, to build a complex with high thermodynamic stability and kinetic inertness, selected BFCA should have high in vivo stability [105]. The complex formed should be in a high yield (>99.5%) with high specific activity. The organo-metallic complex of radiopharmaceuticals should be chemically and biologically stable. The low complex stability will lead to the radiotoxicity induced by transchelation and transmetalation phenomena (see Section 5).

The chemical stability can be observed by their kinetic reaction, the easier complex to form the more stable. The kinetics of complex formation is the function of time and temperature, The long complex formation reaction is unfavorable for the reaction of radionuclides with short half-lives. The temperature that is too high also damages carrier biomolecules. Ideally, a chelator–radionuclide complex is formed in less than 15 min under mild conditions or at room temperature or currently called as a “click reaction” process. The thermodynamic stability of metal complexes is usually expressed in terms of the thermodynamic stability constant (KML, formulated in Equations (1) and (2)). A high thermodynamic stability constant (LogKML > 18) indicates that the complex formed is a stable complex [105,106].


(1) $$M^{m+}+\mathrm{nL}\leftarrow \to {{\mathrm{ML}}_{n}}^{m+}$$

(2) $$K_{\mathrm{ML}}=\frac{\left[ML_{n}{}^{m+}\right]}{\left[M^{m+}\right]\left[L_{n}\right]}=\frac{Kforward}{Kreverse}$$

Another parameter to justify chemical stability of the complex is by observing the magnitude of HOMO-LUMO gap. Highest occupied molecular orbital (HOMO) and Lowest unoccupied molecular orbital (LUMO) are commonly called the frontier orbitals e.g., in the frontier molecular orbital theory. The magnitude gap of energy between these two frontier orbitals reflects the strength and stability of transition metal complexes, as well as the colors they produce in the solution.

Biological stability of radiopharmaceuticals is indicated by the biological efficacy of the radionuclide complex, shown by its biodistribution properties The result of several studies exhibited the same radionuclide had different biodistribution when complexed with different BFCAs. This indicates that the biological efficacy and biodistribution properties depend on the overall structure of radiochemicals formed [104,107,108]. The development of BFCA is in line with the development of radiopharmaceuticals which is very beneficial for the development of radiopharmaceutical synthesis process. The types of BFCAs currently used can be seen in Table 4.

(a) DOTA

DOTA (1,4,7,10-tetraazacyclododecane-1,4,7,10-tetraacetic acid) is the most widely known BFCA that used in radiopharmaceutical research and development [105,109]. DOTA can form complexes with several trivalent metal ions and show high affinity for various types of radionuclides such as ^111^In,  ^86/90^Y, ^44/47^Sc, ^212/213^Bi, ^68^Ga, and ^177^Lu (Table 4) [105,110]. Radionuclides that are bound in the DOTA cavity will remain at room temperature. However, the radiometal can release from the complex to be a free form ion in the body which reduce the concentration of radiopharmaceutical complex in the body as the reverse complexation reaction cannot occure in the room temperature [110]. DOTA complexes with several radionuclides have high thermodynamic stability constant (Table 4) and are also known to have excellent in vivo stability [109,110,111,112,113]. In general, DOTA forms complexes with several radionuclides under room temperature conditions until heated to 100 °C and with a pH range of 4.0–6.0 in 5–30 min [109]. The Zr–DOTA complex whose crystal structure was first reported showed excellent in vivo stability in mice [114,115].

(b) TCMC

TCMC, 2,2,2,2-(1,4,7,10-tetraazacyclododecane-1,4,7,10-tetraacetamide, is a DOTA analogue designed and synthesized to be complexed with ^203^Pb radionuclides [105,109]. The ^203^Pb complexed with DOTA dissociates at pH 7, making it to have acid lability of the complex as a risk of radiotoxicity in metabolic processes. The radiolabeling process with ^203^Pb took place under the conditions of a temperature of 37 °C and pH 5.0–6.5 for 30–60 min. The result of ^203^Pb complexation with TCMC produced ^203^Pb[TCMC] complex that was more resistant to dissociation at acidic pH (pH 3.5 or below) [105,119,120,121]. The TCMC complex with ^203^Pb has a high stability constant with LogK_Pb(TCMC)_: >19 (Table 4) [109,116,117,118].

(c) DOTA coupled Somatostatin Analogs

DOTATATE (1,4,7,10-tetraazacyclododecane-1,4,7,10-tetraacetic acid coupled Tyr3-octreotate), DOTA-NOC (DOTA coupled Nal3-octreotide), and DOTATOC (DOTA coupled Tyr3-octreotide) are the examples of DOTA coupled with a somatostatin analog [122,123]. DOTATATE and DOTANOC exhibit a high affinity for the somatostatin receptor [124,125]. Radiolabeled DOTATATE and DOTANOC reached >99% radiochemical purity and high stability following an easy and convenient protocol. [122]. DOTATOC also shows a high affinity for the somatostatin receptor, high hydrophilicity, and readily forms stable complexes with ^111^In and ^90^Y [123,124]. DOTATOC exhibits good pharmacokinetic characteristics, confirmed by blood clearance and rapid urinary elimination [123]. Radiolabels DOTATATE, DOTATOC, and DOTANOC are currently widely used in treating neuroendocrine tumors due to their high stability and favorable pharmacokinetics and biodistribution properties [122,123].

(d) DTPA

DTPA (diethylenetriaminepentaacetic acid) is an octadentate polyaminocarboxylate acyclic chelator which has long been used in the radiopharmaceutical field. DTPA coordinates with radionuclides such as ^111^In, ^90^Y, ^177^Lu, ^64^Cu, and ^68^Ga (Table 4) by five oxygen donor atoms resulting from deprotonation of the carboxylate group and three nitrogen donor atoms from tertiary amine groups [106]. Like other acyclic BFCAs, DTPA forms a complex with radionuclide at room temperature (25 °C) and pH 4.5–5.5 for several minutes (about 5–20 min), generating stable complexes with radionuclides expessed by higher LogK_ML_ (see Table 4) [105,106,109]. Acyclic BFCAs have problems in vivo stability due to dissociation and transchelation of radiometal complexes. In general, DTPA is not as stable as macrocyclic BFCAs (Ex: DOTA and NOTA), thus leading to the needs for the development of DTPA derivatives [126,127].

(e) 1B4M-DTPA

1B4M-DTPA, 2-(4-isothiocyanatobenzyl)-6-methyl-diethylenetriaminepentaacetic acid is a derivative of DTPA with the addition of methyl and a p-isothiocyanatobenzyl group on the ethylene backbone of DTPA. The in vivo stability of the ^111^In and ^90^Y radionuclide complexed with 1B4M-DTPA has been increased due to the addition of a methyl group that increases the rigidity of the chelator backbone [3,105,109,129].

(f) CXH-A”-DTPA

Another derivative of DTPA is CHX-A”-DTPA, [(R)-2-amino-3-(4-isothiocyanatophenyl) propyl]-trans-(S, S)-cyclohexane-1,2-diamine-pentaacetic acid which built by the addition of a cyclohexane group at the ethylene backbone. This cyclohexane group increases the rigidity of the chelator backbone, thus leading to increased kinetic inertness [105,109]. CHX-A”-DTPA showed a significant increase in stability compared to DTPA, but the stability of CHX-A”-DTPA was still less stable than DOTA. CHX-A”-DTPA has been reported to have been used for several radionuclides such as ^111^In, ^90^Y, ^177^Lu, and ^212/213^Bi (Table 4) [109,130]. Several studies have shown that CHX-A”-DTPA-based radiopharmaceuticals complexed with ^111^In, ^90^Y, ^177^Lu, and ^212/213^Bi have better stability than DTPA and 1B4M-DTPA [131,132]. Radiolabeling of CHX-A”-DTPA and radionuclides occur under room temperature conditions and pH of 5.0–5.5 in 10–30 min [109]. The increased stability of CHX-A”-DTPA compared to DTPA and 1B4M-DTPA establishes CHX-A”-DTPA as being a possible suitable chelator for clinical applications. Baur, et al. (2014) showed that the radiochemical yield of labeling CHX-A”-DTPA with radionuclides ^68^Ga, ^90^Yu, and ^177^Lu reached more than 95% at room temperature and moderate pH values. In addition, the results of the biological activity test also showed high biological activity and good stability in human serum [133].

(g) NOTA

One of the well-known macrocyclic polyaminocarboxylate BFCAs is NOTA (1,4,7-triazacyclononane-1,4,7-triacetic acid). NOTA is a BFCA with small bonding cavity, which is suitable as a chelator for small radionuclides such as gallium and copper [106,109,127,135]. NOTA is widely used as the gold standard for radiolabeling with ^68^Ga because it has great in vivo stability and provides an easy and fast complexation process at room temperature and moderate pH for 30–60 min [106,109]. The thermodynamic stability constant of the ^68^Ga complex with NOTA showed a high value (Table 4) [127,134]. The ^68^Ga complex with NOTA exhibits an octahedral coordination geometry, high thermodynamic stability, and good in vivo stability results in plasma. The results of the study showed that the radiochemical yield of the ^68^Ga-NOTA complex reached more than 95% at room temperature and pH 3 within 10 min [109,134,136]. The ^68^Ga-NOTA complex is also stable in human plasma for 4.5 hours at body temperature (37 °C). Besides being stable to the ^68^Ga, NOTA also showed better stability to form complexes with ^64^Cu when compared to other BFCAs such as DOTA, TETA, DTPA, and EDTA. In the ^64^Cu–DOTA complex, the rate of loss of ^64^Cu from the complex depends on the Cu^2+^ concentration and the pH value. The higher concentration of Cu^2+^ and the increasing pH value will accelerate the loss rate of ^64^Cu. Meanwhile, the rate of loss of ^64^Cu from the ^64^Cu–NOTA complex was minimal depending on the concentration of Cu^2+^ and pH [137,138]. The ^64^Cu–NOTA complex has high thermodynamic stability constant (LogK_ML_ = 21.6) and radiolabeling occurred at room temperature and pH 5.5–6.5 for 30–60 min [109,139]. Zhang et al. compared the in vivo stability of the ^64^Cu–DOTA complex with ^64^Cu–NOTA conjugated with monoclonal antibodies. The results showed that the ^64^Cu–NOTA complex had better in vivo stability indicated by lower liver uptake levels without reducing efficiency to target organs [139].

(h) NODAGA

NODAGA, 2-(4,7-bis(carboxymethyl)-1,4,7-triazonan-1-yl)pentanedioic acid, is one of NOTA derivatives that has been widely developed. NODAGA is widely used to form chelates with ^64^Cu with almost the same thermodynamic stability as NOTA (Table 4) [109]. Several preclinical studies on ^64^Cu–NODAGA bioconjugate were carried out and the results showed an improvement in vivo stability and pharmacokinetic properties. 

A study by Rylova et al. compared the in vitro and in vivo performance of ^64^Cu–NODAGA and ^64^Cu–DOTATATE bioconjugates. The results showed that ^64^Cu–NODAGA had better pharmacokinetic and biodistribution properties than ^64^Cu–DOTATATE bioconjugate. Better biodistribution is indicated by the results of rapid blood clearance and an increase in uptake to target organs [140]. Another study by Ghosh et al. comparing ^64^Cu–DOTA and ^64^Cu–NOTA immunoconjugates showed increased stability in vivo. ^64^Cu–NODAGA immunoconjugates also showed higher cellular uptake. From the results of biodistribution, ^64^Cu–NODAGA immunoconjugates showed lower accumulation in the liver and higher blood activity [141]. The results of preclinical tests showing increased stability to the ^64^Cu–NODAGA complex make NODAGA widely considered for use in clinical applications [109,140,141].

(i) NODASA

NODASA, 2-(4,7-bis(carboxymethyl)-1,4,7-triazonan-1-yl) succinic acid, is a NOTA derivative that was synthesized for radiolabeling with gallium. The stability constant of ^68^Ga-NODASA (Table 4) is not much different from the value of the stability constant of ^68^Ga-NOTA (Table 4) [109,142,143]. The in vivo stability of ^68^Ga-NODASA in blood serum showed that there was no transchelation with transferrin in blood serum for five days of observation; it can be said that ^68^Ga-NODASA had high in vivo stability. In addition, the stability of the ^68^Ga-NODASA complex was also observed under environmental conditions of pH 2 and temperature of 37 °C. The results showed that the ^68^Ga-NODASA complex remained 100% for five days of observation. The high stability of the ^68^Ga-NODASA complex promises to produce stable radiopharmaceutical agents [109,134,142,143].

(j) NETA

In addition to the derivative of NOTA, the analog of NOTA, NETA, 2,2′-((2-(4,7-bis(carboxymethyl))-1,4,7-triazonan-1-yl)ethyl)azanediyl)diacetic acid, has also been reported for radiolabeling with several radionuclides such as ^177^Lu, ^90^Y, and ^205/206^Bi (Table 4). NETA is reported to have both acyclic and cyclic chelator characteristics. The cyclic components provide thermodynamic stability and rigidity to the complex, while the acyclic components of NETA can accelerate the complexation process. Complexation occurred at room temperature and pH 4.0–4.5 in 5 min [109,146]. The efficiency of NETA radiolabeling for several radionuclides ^177^Lu and ^90^Y showed high efficiency and maximum specific activity under mild conditions (more than 99%). The results of the ^177^Lu and ^90^Y complexed with NETA also showed in vivo stability in human serum by showing no radionuclide loss during 14 days of observation. In addition to in vivo and kinetic stability, the pharmacokinetic profile of the radionuclide complex with NETA has improved pharmacokinetic properties [144,145,146].

(k) TETA

Another macrocyclic BFCA besides DOTA and NOTA is TETA (1,4,8,11-tetraazacyclotetradecane-1,4,8,11-tetraacetic acid), which has been extensively studied to form chelates with copper. DOTA and TETA have an octadentate coordination shape, but the ring size of the TETA structure is larger than that of DOTA [105,109]. TETA was able to form complexes with ^64^Cu at room temperature and environmental pH in the range of 5.0–7.0 for 60 min. The value of the stability constant (LogK_ML_) of the ^64^Cu-TETA complex is 21.9 (Table 4) [147,148]. The ^64^Cu-TETA complex was reported to have low kinetic inertness and was unstable in vivo due to the transchelation of Cu^2+^. The results of the transchelation test of the ^64^Cu-TETA complex showed that Cu^2+^ could dissociate from the complex and bind to high amounts of protein. Currently, TETA is not used to form chelates with ^64^Cu due to the poor biochemical stability [149,150].

(l) CB-TE2A

The development of the TETA analog to overcome the stability problem of TETA was carried out and a bicyclic analog of TETA, which has an ethylene cross-bridge, was obtained, namely, CB-TE2A (2,2-(1,4,8,11-tetraazabicyclo[6.6.2]hexadecane-4,11-diyl)diacetic acid). ^64^Cu-CB-TE2A showed less amount of ^64^Cu bound to protein (13%) compared to ^64^Cu-TETA (75%) during 4 h of observation [151,152]. CB-TE2A also showed less transchelation than TETA. The presence of an ethylene cross-bridge increases the stability of the complex and prevents the transchelation of ^64^Cu. CB-TE2A shows better potency for application in radiopharmaceuticals. However, the disadvantage of using CB-TE2A is that it requires heating up to 90 °C to carry out the radiolabeling process for 1 h. Excessive heating might damage the biomolecules used in the synthesis of radiopharmaceuticals [109,151,152,153,154].

(m) H_2_dedpa

The BFCA group called “pa family” is a type of chelator derived from picolinic acid, which developed as an alternative to polyaminopolycarboxylic acid derivative ligands [155,156,157]. H_2_dedpa is the first known chelator in the “pa family”. H_2_dedpa is an acyclic hexadentate chelator used to form complexes with gallium. The H_2_dedpa complex with gallium occurs under room temperature conditions in a short time of about 5–10 min. The radiochemical yield of Ga(III)-dedpa complex is more than >99% with high specific activity without any purification step. The Ga(III)-dedpa complex was also thermodynamically stable with LogK_ML_ = 28.1 [155,158,159]. The high value of the thermodynamic stability constant indicates the high affinity of H_2_dedpa for gallium. The Ga(III)-dedpa complex had high in vivo stability and was not transchelated with apo-transferrin in human serum after two hours of observation [155].

(n) H_4_octapa

The derivative of H_2_dedpa, H_4_octapa was synthesized by adding a carboxylic acid arm to the H_2_dedpa structure. H_4_octapa was reported to form a fast and stable complex with ^111^In (LogK_ML_ = 26.8) and ^177^Lu (LogK_ML_ = 20.1) (Table 4). H_4_octapa was able to radiolabel with ^111^In and ^177^Lu at room temperature and pH 4.5 in a short time of about 5–10 min [160,161]. The in vitro stability of the ^111^In-octapa complex in mouse serum showed better stability compared to DOTA and DTPA after 24 h. The good in vivo stability of the ^111^In-octapa complex showed by the results of the biodistribution test (lower uptake occurred in the liver, pancreas, and kidneys) [161]. Conjugation of the ^117^Lu-octapa complex with antibody was carried out to determine in vivo stability. Conjugation between H_4_octapa with antibody proceeds rapidly under mild conditions with a high yield (94–95%) compared to antibody conjugation with DOTA (50–80%). The in vivo stability of conjugated H_4_octapa showed an improved biodistribution profile and imaging results using SPECT [160].

(o) H_2_CHXdedpa and H_4_CHXoctapa

To improve the stability of H_2_dedpa and H_4_octapa, chelators H_2_CHXdedpa and H_4_CHXoctapa were synthesized to be labeled with ^67^Ga and ^111^In. H_2_CHXdedpa was radiolabeled with gallium and showed high radiolabeling efficiency (more than 99%) under room temperature for 10 min. The stability of the Ga-CHXdedpa complex carried out in the presence of apo-transferrin at 37 °C showed that the complex still remained around 86–99% after 2 h of observation [162]. The Ga-CHXdedpa complex exhibited similar properties to Ga-dedpa and showed high thermodynamic stability (LogK_ML_ = 28.11) [115,157]. The results of in vivo kinetic inertness showed that Ga-CHXdedpa was more stable than Ga-dedpa. The results of transchelation testing in human serum showed that 90.5% of Ga-CHXdedpa remained after 2 h, whereas only 77.8% of Ga-dedpa remained [157]. The In-CHXoctapa complex (LogK_ML_ = 27.16) showed a higher stability constant than the In-octapa complex (LogK_ML_ = 26.76) [163,164]. The In-CHXoctapa complex also showed high kinetic inertness values in human serum after 120 h compared to H_4_octapa and standard BFCAs such as DOTA and DTPA [157].

(p) HYNIC

HYNIC (hydrazinonicotinamide) is a chelator used to form chelates with ^99m^Tc and ^186^Re. The HYNIC structure consists of a hydrazine ligand combined with an aromatic nitrogen donor to create a bidentate chelator. The carboxylate group in the HYNIC structure serves to conjugate chelators with biomolecules through amide bonds [105]. Since it was first reported that HYNIC can form chelates with ^99m^Tc, now HYNIC is widely used to label antibodies or biomolecules with ^99m^Tc. The ^99m^Tc labeling process required heating to a temperature of 100 ^o^C for 20–30 min. The HYNIC complex with ^99m^Tc formed has high purity (>95%), low radiochemical yield (<90%), and high specific activity (>1 Ci/mmol) [165,166]. In the process of radiolabeling HYNIC with ^99m^Tc, it is necessary to add a co-ligand to complete technetium coordination [166]. Co-ligands include EDDA (ethylenediamine diacetic acid), aromatic amines, aminothiols, water-soluble phosphine, and tricine. However, the addition of co-ligands to the radiometal-HYNIC complex can lead to stereoisomer formation that is difficult to characterize and exhibit different pharmacokinetic properties [165].

(q) EDDA/HYNIC-TOC

EDDA/HYNIC-TOC is a derivative of HYNIC, which shows excellent in vitro and in vivo stability. Decristoforo et al. compared ^99^m-Tc-EDDA/HYNIC-TOC with ^111^In-DTPA-octreotide ^111^In-DOTA-TOC to diagnose various cancers or tumors (thyroid cancer, carcinoma syndrome, pancreatic cancer, and pituitary tumor). ^99^mTc-EDDA/HYNIC-TOC can be imaged within 15 min after injection and reach the maximum ratio in target or non-target organs within 4 h after injection. The blood clearance rate is faster than ^111^In-DOTA-TOC. The study results concluded that ^99^m-Tc-EDDA/HYNIC-TOC could be used for diagnosis because it has good imaging properties [168].

(r) Sar Chelator

Sar or sarcophagine (3,6,10,13,16,19-hexaazabicyclo[6.6.6]icosane) was first developed in 2001 by Sargeson to form stable complexes with copper [105,169]. The Sar group chelator is a hexaazamacrobicyclic ligand and consists of SarAr, SarAr-NCS, diamSar, AmBaSar, and BaBaSar [170,171,172,173]. Radiolabel Sar with ^64^Cu occurs at room temperature in a short time, around 5–30 min. The rapid radiolabeling process is an advantage of the Sar group chelator because it contains many nitrogen atoms [7,109,169]. Sar coordinates with ^64^Cu to form a cage structure through multiple macrocyclic rings so that the complex formed is stable and resistant to dissociation [107,118,172]. The Sar chelator can overcome the problem of the in vivo lability of ^64^Cu, so the ^64^Cu-Sar complex was first used for imaging neuroblastoma and melanoma. Radiolabeled Sar with ^64^Cu gave a radiochemical yield of more than 95% with a specific activity of about 10mCi/mg. Biodistribution results showed lower ^64^Cu-Sar accumulated in the liver (5–10%) [170]. The ^64^Cu-Sar complex underwent <6% dissociation after 48 h in human serum. Radiolabeling Sar with ^64^Cu achieves a radiochemical purity of over 99% with an efficiency of about 98%. The ability of Sar to form stable complexes with ^64^Cu is a consideration for the widespread use of Sar in radiopharmaceuticals for diagnosis using PET [171,172,173].

(s) T_3,4_BCPP

In addition to acyclic and macrocyclic chelators, porphyrin-type chelators have been widely developed for application in radiopharmaceuticals and biomedical sciences [175]. It was initially reported that the chelator T_4_CPP (5,10,15,20-tetrakis[4-(carboxymethyleneoxy)phenyl]porphyrin) accumulates selectively in mammary tumor-bearing rats and sarcoma tumor-bearing mice. Then, a water-soluble porphyrin was developed, namely, T_3,4_BCPP (5,10,15,20-tetrakis[3,4-bis(carboxymethyleneoxy)phenyl]porphyrin), which was later patented by the Indian Patent Office [177,178]. T_3,4_BCPP was also reported to accumulate more in tumor cells compared to normal tissue or cells. Several studies have shown that T_3,4_BCPP can form complexes with ^99m^Tc and ^188^Re [175,176,177]. Radiolabeling of T_3,4_BCPP with ^99m^Tc was carried out at pH 8, and the purity of ^99m^Tc-T_3,4_BCPP was over 95%. ^99m^Tc-T_3,4_BCPP was also stable in vitro for 4 h at room temperature. The 99mTc-T3,4BCPP complex has good in vivo performance indicated by the lack of affinity of the complex to the stomach and thyroid where free pertechnetate accumulates. The efficiency of the T_3,4_BCPP radiolabel with ^188^Re reached 98.2%. The radiochemical yield of ^188^Re-T_3,4_BCPP reached 95%, but the radiochemical yield reached more than 98% if the radiolabeled pH was in the range of 1.5–5.0 and heated to a temperature of 95–100 for 30 min. The in vitro stability of ^188^Re-T_3,4_BCPP in saline and serum reached more than 95%, indicated by a significant change in the number of ^88^Re-T_3,4_BCPP during 48 h of observation at room temperature. The biodistribution results showed low activity of ^88^Re-T_3,4_BCPP in non-target organs and tissues after 24 h of injection. Due to the good properties and resistance of ^99m^Tc-T_3,4_BCPP and ^88^Re-T_3,4_BCPP, the complexes have been considered as tumor imaging agents [175,176].

(t) N(NOEt)_2_

In an advance in the diagnostic process using the radionuclide technetium-^99^m, the ligand (Ethoxy(ethyl)amino)methanedithiol is used to form a complex with the radionuclide ^99m^Tc in the radiopharmaceutical diagnosis process. It has been reported that N(NOEt)_2_, bis(N-ethoxy-N-ethyldithiocarbamato)nitride, was used as an imaging agent for myocardial perfusion after radiolabeling with ^99^mTc [180]. Currently, isomers of N(NOet)_2_ are being developed, namely, anti, syn-endo, and syn-exo, to determine which isomer is more stable than N(NOEt)_2_. The stability, reactivity, and structural properties of the three ^99^m-TcN(NOEt)_2_ isomers were compared. The stability and reactivity of ^99^m-TcN(NOEt)_2_ were carried out by the compatibility method, and the results were expressed in terms of the molecular orbital energy gaps (HOMO and LUMO) [180]. The lower the HOMO–LUMO energy gap indicates the more reactive compounds (having high reactivity). Of the three ^99^m-TcN(NOEt)_2_ isomers, anti^99^mTc-N(NOEt)_2_ has the lower HOMO–LUMO gap. Hence, it can be the most reactive among the other three isomers. All three isomers form a stable complex with ^99^m-Tc, but anti-^99^mTc-N(NOEt)_2_ can be stated as the most stable isomer [180,181].

(u) HBED-CC

N,N’-bis[2-hydroxy-5-(carboxyethyl)benzyl]ethylenediamine-N,N’-diacetic acid (HBED-CC) is an acyclic BFCA commonly used to form complexes with gallium. HBED-CC has high thermodynamic stability with Ga (LogKML = 38.5) and can form complexes rapidly due to its acyclic structure [109,182,183]. Radiolabeled HBED-CC with ^68^Ga occurred at room temperature and a pH range of 4–4.5 for 10–20 min. The HBED-CC complex with ^68^Ga showed great in vivo stability. The radiochemical purity of the ^99^mTc-HBED-CC complex reaches more than 95% with high in vitro stability. In vitro stability of the ^99^mTc-HBED-CC complex was demonstrated by no transchelation when the complex was assayed against L-histidine or L-cysteine at 37 [182,183].

(v) PCTA-NCS

Radionuclide ^177^Lu is widely used in radiopharmaceuticals for cancer therapy. Several types of BFCA are used to form complexes with ^177^Lu, such as DOTA and DTPA [144,184,185]. Although DOTA and derivatives have high efficiency for use as a ^177^Lu chelator, the complex formation process takes a long time at room temperature. The formation of the DOTA complex with ^177^Lu requires heating, which can damage biological molecules [186]. Therefore, the PCTA-NCS ligand was developed as a suitable chelator for ^177^Lu. The radiolabeling yields of ^177^Lu-PCTA-NCS reached more than 95% in less than 15 min at room temperature. In vitro stability studies of ^177^Lu-PCTA-NCS showed that more than 95% of the ^177^Lu-PCTA-NCS complex remained in saline and serum after 7 days of incubation. Most of the ^177^Lu-PCTA-NCS complex remained in tissue and organs after 24 h of injection. In addition, the ^177^Lu-PCTA-NCS complex did not show any significant uptake in some vital organs such as the liver. PCTA-NCS can be used for radiolabeling with temperature-sensitive biomolecules within a few minutes at room temperature [185].

(w) MANOTA

Improved BFCA is needed for monoclonal antibody radiolabel with ^64^Cu under mild conditions. Moreau et al. showed that MANOTA (Methyl AminotriazacycloNOnane Triacetic Acid) is a BFCA used for radiolabeling ^64^Cu with monoclonal antibodies for immunoPET imaging. DOTA-, DOTAGA-, NOTAGA-, and MANOTA-containing isothiocyanate grafting functions were conjugated with monoclonal antibodies. BFCAs conjugated with a monoclonal antibody labeled with ^64^Cu at 37 °C in 5 min. The radiolabel results gave a radiochemical yield of more than 97%. MANOTA showed significantly higher stability for 48 h (>95%) compared to DOTA, DOTAGA, and NOTAGA (88–91%). The stability of MANOTA in plasma showed a yield of more than 97%. The ^64^Cu-MANOTA complex was not transchelated in the presence of EDTA, indicating high stability of the complex. In vivo stability of ^64^Cu-MANOTA immunoconjugate was demonstrated by low uptake from non-target organs and a high ratio of ^64^Cu-MANOTA in target organs [187].

(x) THP

Tris 3,4-hydroxypyridinone (THP) is a non-aminocarboxylic acid chelator used for ions such as Ga^3+^ and Fe^3+^. THP is able to form complexes with Fe^3+^ at low Fe^3+^ ion concentrations with constants (LogK = 14.2) [188,190]. Radiolabeling of THP with ^68^Ga occurred at room temperature for 5 min, being one of the advantages of THP for radiolabeling ^68^Ga [189]. A THP-functionalized bioconjugate labeled with ^68^Ga has been developed. THP-TATE was radiolabeled with ^68^Ga and the radiolabeling process was fast (less than 2 min). The radiochemical yield was more than 95% at room temperature with a specific activity of 60–80 MBq nmol^−1^. In vivo study of ^68^Ga(THP-TATE) showed that less than 2% of Ga^3+^ transchelated serum proteins during 5 h of observation in human serum (37 °C). High in vivo stability is indicated by the accumulation of ^68^Ga(THP-TATE) specifically in target organs. A favorable biodistribution profile was shown by ^68^Ga(THP-TATE) [191]. In addition to the study of THP-TATE, a study of THP-PSMA radiolabeled with ^68^Ga was also carried out. THP-PSMA is THP conjugated with a single-chain antibody against prostate-specific membrane antigen (PSMA). Radiolabeled THP-PSMA for 5 min at room temperature and neutral pH. The radiochemical yield obtained is about 97%. Results of stability studies of ^68^Ga(THP-PSMA) in human serum (37 °C) showed neither Ga^3+^ loss nor transchelation to serum proteins [86] THP was currently identified as a chelator used for the development of ^68^Ga radiolabeling kits [191,192].

(y) DFO, DFO*, and DFOcyclo*

DFO (desferrioxamine) is an acyclic hexadentate chelator that is a competent chelator for radiolabeling with ^89^Zr [113,114]. DTPA has been reported to form a thermodynamically stable complex with ^89^Zr, but several studies have reported poor in vivo stability of the ^89^Zr-DTPA complex [109]. DFO was able to form a stable complex with ^89^Zr at room temperature and the radiochemical yield reached >99% within 1 h [109]. DFO exhibits a fast and efficient radiolabel. The resulting complex had good stability and less than 0.2% of ^89^Zr was released into the serum for 24 h. So far, the thermodynamic stability of the ^89^Zr-DFO complex has not been reported. [113,114,193]. Currently, DFO is conjugated with antibodies by several methods. Different methods and techniques for conjugating DFO with antibodies may affect the efficiency and biodistribution profile of the conjugated products [113,114]. As a hexadentate chelator, DFO is not able to form perfect coordination with ^89^Zr. The ^89^Zr-DFO complex is susceptible to dissociation and causes the accumulation of free ^89^Zr in bone [193].

The analog of DFO was developed as an octadentate chelator, which forms a complex with ^89^Zr more stable than that of DFO. The ^89^Zr complex with DFO* is also more resistant to transcription than DFO. Complexes ^89^Zr-DFO and ^89^Zr-DFO* conjugated with trastuzumab were then evaluated for in vivo and in vitro stability. Bone uptake in the ^89^Zr-DFO* complex was significantly lower than that in ^89^Zr-DFO. This result shows that ^89^Zr-DFO* has better in vivo and in vitro stability [194,195]. Zr-DFO complexes have poor in vivo stability, complexes undergo radiometal dissociation, and lead to free ^89^Zr accumulation in the bone. DFO* is an analog of DFO. DFO* improves in vivo stability of ^89^Zr-DFO* complex and increases the stability of complex transchelation compared to DFO [195].

DFOcyclo* properties are more lipophilic than DFO and DFO* (log D 2.14 ± 0.10). Conjugation between DFO, DFO*, and DFOcyclo* with trastuzumab showed DFOcyclo* had a higher ratio of conjugation (conjugation ratio of DFO, DFO*, and DFOcyclo* with trastuzumab sequentially 2.6, 2.6, and 3.7). DFO* and DFOcyclo* complexes with ^89^Zr showed greater in vivo stability than ^89^Zr-DFO complexes in the presence of excess EDTA [196].

## 4. Pharmacokinetic-Modifying Linker Used in Radiopharmaceutical Agents

In radiopharmaceuticals, linker’s aim to modify radiopharmaceutical’s pharmacokinetics, known as pharmacokinetic-modifying linkers (PKM linkers) [103,104,105]. PKM linkers can improve the “target-to-background” ratio by modifying the pharmacokinetic properties (distribution or excretion) of radiolabeled biomolecules. In addition, PKM linkers can reduce the accumulation of substances in non-target organs and increase the rate of uptake from target organs [103,104,197]. PKM linkers commonly used are PKM linkers made from polyethylene glycol, aminohexanoic acid, amino acids, etc. Linkers used in radiopharmaceuticals must be stable under human physiological conditions and have no effect or affinity on target organs [105]. Linkers commonly used in radiopharmaceuticals are listed in Table 5.

(a) EGS and DSS

Quadri et al. showed improvement in the pharmacokinetic properties of EGS (ethylene glycol bis(succinimidyl succinate) and DSS (disuccinimidyl subera) immunoconjugate. EGS immunoconjugate showed clearance from the blood and faster excretion. In addition, EGS immunoconjugates also showed improvement in tumor targeting. Stability studies of DSS immunoconjugate in 30–40 mCi/animal of dose injection displayed no colloid formation, and serum stability in 24 h (92–97%) 48 h (85–90%). The complex had 85% immunoreactivity. The result showed that both EGS and DSS were compatible with the chelator and targeting vector [197].

(b) EMCS-Bz, MESS-Bz, and MIH

EMCS-Bz-EDTA, MESS-Bz-EDTA, and MIH-EDTA were coupled with the monoclonal antibody OST7. OST7 MIH had slightly faster clearance than ECMS. On the other hand, MESS-Bz had a faster clearance in biodistribution studies. OST7-MESS-Bz-EDTA released radioactivity rapidly, and more than 95% of the radioactivity was released over 24 h. OST7-MIH released about 20% radioactivity. The stability of the ester bond in the radioimmunoconjugate affected the structural changes in the linker and the radiolabel attached to the monoclonal antibody. EMCS-Bz-EDTA and MESS-Bz-EDTA with ester bonds showed higher stability than that of MIH [198].

(c) N4

The study results show the properties of conjugation of 6-carboxy-1,4,8,11-tetraazaundecane (N4) with several radio substances [199]. The N4-conjugated and ^99^mTc-labeled radiopeptide showed superior properties, with very high cell uptake in vitro and about the highest tumor uptake at 1 and 4 h of any somatostatin-based radiopeptide studied to date in this xenograft model. The addition of N4 showed increased in vivo and in vitro stability and pharmacokinetic properties [203,204].

(d) p-aminomethylaniline-diglycolic acid

p-aminomethylaniline-diglycolic acid was used as a linker in the GRPR-antagonist SB3, labeled with various radionuclides, such as ^68^Ga, ^111^In, and ^177^Lu [205]. The p-aminodimethylaniline-diglycolic acid linker can be used to link ^68^Ga-DOTA with D-Phe-Gln-Trp-Ala-Val-Gly-His-Leu-NHEt for PET tracer based on GPCR. PET tracer-based GRPR radio antagonist ^68^Ga-DOTA-p-aminomethyl aniline-diglycolic acid-D-Phe-Gln-Trp-Ala-Val-Gly-His-Leu-NHEt (^68^Ga-SB3) experiment result showed excellent efficacy in tumor localizing and pharmacokinetics in prostate cancer and breast cancer patients [202].

(e) PEG

Polyethylene glycol (PEG) is a non-toxic, non-antigenic, non-immunogenic, and water-soluble polymer. The FDA has approved the use of PEG. The process of modifying proteins and molecules by incorporating one or more PEGs is called PEGylation [203]. The results of conjugation with PEG showed favorable pharmacokinetic properties of the substance. PEGylated radiopharmaceutical peptide base was developed and evaluated. The evaluation results showed an increase in molecular uptake in the target organ and a decrease in molecular uptake in the kidney, a longer circulation rate, an increase in water aqueous solubility, and in vivo stability [203]. The results showed that the incorporation of PEG into the complex could increase the stability of the complex and be excreted rapidly. Experimental studies were carried out by conjugating the PEG linker with ^89^ZrDFO. The results show that the addition of a PEG linker improved radiochemical conversion and aqueous phase solubility [204].

## 5. Radiotoxicity Based on Clinical Setting

It is well understood that toxicity can be linked to a radiopharmaceutical of radiation doses. Desired diagnostic usage by radiopharmaceuticals has consequences and also desired characteristics of radiopharmaceuticals for use in treatment. Although they have safety records, some things can lead to toxicity in radiopharmaceuticals, such as in vivo instability, very poor tumor penetrability, slower circulatory clearance, and accumulation in non-specific sites [206]. Instability can occur in case radiopharmaceuticals are labeled with radiometals (e.g., ^68^Ga,^99m^Tc, and ^64^Cu). In vivo toxicity of free radiometal is a major concern. Therefore, a proper design should be carefully considereded to minimize radiometal detachment from chelating agent. The peptide was promptly degraded and eliminated from the body in some instances, increasing background radiation, which lowers imaging sensitivity [207]. There are several external and in vivo elements that might affect radiopharmaceutical biodistribution. Some of the factors that cause this change are blood transfusion, renal clearance, radiopharmaceutical formulation, and a strong deposit at a specific location [208].

(a) Radiotoxicity induced by carrier molecule’s activity

Prostate-Specific Membrane Antigen (PSMA) is currently used as a target in developing radiopharmaceutical agents for imaging and treatment of prostate cancer because about 90–100% of prostate cancer cases experience PSMA overexpression [209]. PSMA-targeting molecules are labeled with radionuclides that will provide radiation to prostate cancer cells. The molecule that was first approved for use to target PSMA by the FDA was the monoclonal antibody 7E11 [210]. However, PSMA is not only expressed in prostate cancer or prostate tissue. PSMA is also expressed in several other tissues such as salivary glands, kidneys (proximal tubular cells of the nephron), and small intestine (duodenum). PSMA targeting molecules can be distributed in healthy tissues and potentially cause toxicity in healthy tissues [206,211]. The toxicity of [^177^Lu]Lu-PSMA-therapy has been investigated. The results show that using monoclonal antibodies as carrier molecules can cause hematological and bone marrow toxicity due to the long circulation time of the antibodies in the body [211,212]. Using small molecules as a carrier, healthy organ distribution usually occurs in the kidneys, salivary glands, and lacrimal glands [213]. A study showed that in the kidney, it was reported that 4.5% of patients had mild nephrotoxicity. Approximately 30% of patients receiving [^177^Lu]Lu-PSMA-617 therapy had mild to moderate xerostomia, indicating salivary gland toxicity. Moderate to severe xerostomia was reported following the first phase clinical trial of [^225^Ac]Ac-PSMA-617 [214,215]. Several strategies could be used to protect the salivary glands from toxicity (salivary gland hypofunction and xerostomia), including using ice packs, injecting botulinum toxin, and stimulating salivary secretion. The cooling method using ice packs can reduce the uptake of PSMA target compounds, induce vasoconstriction and reduce blood flow to the salivary glands. Administration of botulinum toxin can suppress the metabolism in the salivary glands. Salivary secretion can be induced by giving lemon water or eating yogurt. Prevention of toxicity to the salivary glands becomes important when performing treatment using PSMA-targeted therapy for prostate cancer [216,217,218].

Somatostatin receptors were frequently overexpressed in neuroendocrine tumors (NETs), and radiolabeled somatostatin analogs are helpful tools for in vivo diagnosis and treatment of NETs. ^68^Ga-DOTATATE and ^68^Ga-DOTATOC are the two most often utilized chemicals in functional imaging with PET. According to Poepple et al., 2015, 68Ga-DOTATOC may be superior to ^68^Ga-DOTATATE, owing to increased tumor absorption as demonstrated by SUVmax (12.7 + 3.0 vs. 13.2 + 3.3). This disparity in affinity profiles might explain why tumor uptake differs. In other cases, the somatostatin receptor, DOTATOC, is targeted with suitable metals ^90^Y and ^177^Lu in peptide receptor radionuclide treatment (PRRT). However, it has significant toxicity to bone marrow and liver. Radiation effects are caused by the presence of somatostatin in the region [219]. Because ^90^Y has a greater energy β particle than ^177^Lu, it may be more successful in treating big tumors. Nevertheless, compared to ^177^Lu-based PRRT, 90Y-based PRRT has been linked to higher marrow toxicity rates. A lower glomerular filtration rate at the time of enrollment was the primary risk factor for renal adverse events [220]. Because nephrotoxicity is significant toxicity in PRRT, ^177^Lu-DOTATATE has more extended residence periods in tumors and kidneys than ^177^Lu-DOTATOC [219]. DOTATOC has lower toxicity to renal parenchyma cells, so the toxicity will be lower than DOTATE. In the use of NETs radiometal, ^177^Lu is safer than ^90^Y for liver cells and kidney cells.

In 2002 and 2003, the FDA approved the use of ^90^Y-ibritumomab (Zevalin) and ^131^I-tositumomab (Bexxar) for use in the treatment of B-cell non-Hodgkin’s lymphoma (NHL). Zevalin and Bexxar target the CD20 antigen, which is more than 90% expressed in NHL [221]. The maximum tolerated dose of Bexxar was 0.75 Gy and was used for a phase II clinical trial of ^131^I-tositumomab. From the results of clinical trials, it was observed that patients who were given ^131^I-tositumomab could experience nonhematologic side effects (16% of total patients) and hematological toxicity (50% of total patients). Clinical trials were also carried out on Zevalin and the most common reaction to administration was cytopenia, followed by infection and inflammation (febrile neutropenia and sepsis). Dosimetry analysis of Zevalin in several clinical trials showed the radiation absorbed dose from Zevalin was still within safe limits and did not correlate with toxicity [222].

(b) Radiotoxicity induced by radionuclide’s properties

Radiotoxicity can be induced due to the intrinsic properties of the radionuclide. Various chemical properties of radionuclides (e.g., electronegativity, oxidation state, etc.) commonly lead to a transmetallation and transchelation between radionuclide and endogenous metal, i.e., Fe^2+^ in hemoglobin, Ca^2+^ in bone, or other metalloenzymes [223,224].

Transmetalation occurs when radiometal in a radiopharmaceutical structure is replaced with an endogenous free-form metal ion in the body (e.g., Ca^2+^, Zn^2+^, Fe^3+^, etc.). The decomplexed-radiometal will be freely distributed in the human body as an ion and generate toxicities in the bone marrow due to the long-term radiation of lanthanide accumulation as well as producing noise to the imaging sensor [217,218]. This has been observed as the important cause of various ^64^Cu toxicity in rats, e.g., a poor liver clearance, and an increasing bone marrow uptake leading to a significant drop in the number of white blood cells [225].

Transchelation is the exchange of chelator (inorganic ligand of the organometallic complex). This transligation process happens when the radiometal is scavenged by a metalloprotein that can naturally build another organometallic complex. This kind of phenomenon occurs if the radiometal has a similar oxidation state or very close ionic radii. The transchelation of ^64^Cu-DOTA to the superoxide dismutase (SOD) in the liver has been identified as a cause of necrotic-hepatotoxicity in vivo [226].

As described above, there is a big need to adjust and control these transmetallation and transchelation phenomena of radiometal-based radiopharmaceutical agents for human use.

## 6. Impact to the Future Trend of Radiopharmaceuticals

Recently, radiopharmaceutical applications have been used for treatment to ensure a bright future in oncology with radioligand theranostics (RT), which includes imaging using positron emission tomography (PET), single-photon emission computed tomography (SPECT), and planar scintigraphy for diagnosis and therapy with personalized management output [227]. A ligand–linker–chelator structure allows for distinct changes in possibly affecting pharmacokinetics and pharmacodynamics. The pharmacokinetics, pharmacodynamics, and biodistribution patterns of radiopharmaceuticals are all affected by changes in the linker and chelator regions. Several nucleotides such as ^47^Sc, ^90^Y, ^131^I, ^166^Ho, ^177^Lu, ^188^Re, and ^213^Bi will be the most regularly manufactured radionuclides because of their capacity for theranostics purposes (for diagnosis and therapy at once) [228]. Development of a strategy for building radiopharmaceuticals using a small molecule [229], amino acid [230], and peptide labeling [116] and using a macro-chelating agent for radiometal labeling can be used in a strategy to construct radiopharmaceuticals with improved stability [108].

Specific radiopharmaceuticals are used in cancer diagnosis, but they are also used in body tracers to track physiology. Non-invasive radionuclide physiologic scanning is an excellent example of the ongoing need for dedicated radiotracers, improved imaging technologies, and more advanced compartmental models to allow for a complete characterization of the metabolism. Some radionuclides are used to identify metabolisms, such as ^123^I, ^11^C, and ^18^I for cardiac diagnostics [231] or Tau PET for Alzheimer’s disease diagnosis [232]. For oncology purposes, different cancer theranostic techniques have been developed in the four summaries. First, direct imaging and quantification of target expression utilizing a single radiolabeled chemical for diagnosis and therapy without changing the target’s expression. The second uses theranostic pairs, which combine two radiopharmaceuticals with the same structure and target but differentially labeled with matching radioisotope pairs that allow diagnosis and therapy to perform separately; indirect imaging using reporter gene technology; and the fourth, imaging downstream effects of gene- and cell-based therapies [233].

## 7. Conclusions

Advancement in nuclear medicine requires a better knowledge of radiopharmaceuticals. As a result, this review has summarized the components of radiopharmaceuticals, which include radionuclides, bifunctional chelating agents, and pharmacokinetic-modifying linkers. A newly designed theranostic drug based on radionuclides with alpha and beta has a longer lifetime for therapeutic purposes than that of gamma energy, for diagnostic purposes. According to energy ionization, gamma emitters have a higher level for therapeutic use but have smaller LET than beta and alpha emitters. Radionuclide for metal theranostics cannot be stable if conjugated with a carrier. Thus, it must be complexed with a bifunctional chelating agent. The critical aspect for the compatible chelating agent is to look at Homo–Lumo value and Log_KML_ > 18. Modified pharmacokinetics can be designed by the pharmacokinetic-modifying linker. PKM linkers can reduce the accumulation of substances in non-target organs and increase the rate of uptake from target organs. For the future strategy, the scaffold can use the design of small carrier molecules, amino acids, and peptides with direct and indirect labeling.

## Figures and Tables

**Table 1:** Physical and biological characteristics of alpha, beta, and gamma radiation.

Parameters	Alpha Radiation	Beta Radiation	Gamma Radiation	References
Energy (MeV)	5–8	0.5–2.3	0.1–0.5	[5,6]
Range in tissues (mm)	0.05–0.08	1–12	33–164	[6]
LET (keV/μm)	60–230	0.1–1.0	0.3	[6,7]
RBE	5–10	0.9	0.8–0.9	[7]
Half-life	1 h–10 d	7 h–7 d	1 m–5 d	[3]
DNA localization effect	Yes	No		[4]
Radiate to non-targeted cells	Yes	No		[4]
Tumor crossfire	No	Yes		[4]

**Table 3:** Radionuclides for therapy purposes.

Radionuclide	Half-Life	Mode of Decay	Energy (KeV)	Indication (in Radiopharmaceutical Form)	References
^90^Y	64.10 h	β^−^ β^+^ γ	2270 (100%) 739 (0.003%) 511 (0.006%)	90Y-microsphere (TheraSphere^®^ and SIR-Spheres^®^) * radiotherapy for hepatic metastasis, 90Y-ibritumomab tiuxetan ** for lymphoma, and ^90^Y-hydroxypatite and ^90^Y-citrate colloid ** for leukemia PVNS (synovitis).	[84,85]
^117m^Sn	13.6 d	IT	130 150	^117m^Sn-DTPA *** for bone tumor treatment and palliative therapy.	[84]
^131^I	8.02 d	β^−^; γ	606 (89.3%); 364 (81.2%)	^131^I (radioactive iodine therapy) * use for therapy in thyroid cancer, for hyperthyroidism, RIT for NHL, and therapy for malignant pheochromocytoma neuroblastoma	[84,86]
^153^Sm	46.5 h	β^−^	808 (20%); 710 (50%)	^153^Sm-EDTMP * for painful bone metastasis and synovitis tratment.	[84,85,87,88]
^177^Lu	6.73 d	β^−^	498 (78%)	^177^Lu-HA **** for synovitis treatment, ^177^Lu-PSMA-617 (Pluvicto) * for prostate cancer, ^177^Lu-DOTATATE (Luthatera ^®^) * for neuroendocrine tumor.	[84,85]
^225^Ac	10 d	α	5793 (18.1%) 5830 (50.7%)	^225^Ac-PSMA-617 **** for prostate cancer, ^225^Ac-lintuzumab *** for leukemia, and ^225^Ac-NOTA-trastuzumab ***** for breast cancer treatment	[89]
^186^Re	3.72 d	EC, β^−^	1965 β^−^ (25.6%)	^186^Re-HEDP *** for painful skeletal metastasis and painful arthritis	[84,85]
^188^Re	17.00 h	β^−^, γ	2120 (71.1%)	^188^Re-HEDP *** for painful bone metastasis, rheumatoid arthritis, and treatments for RIT with various cancers	[84,85]
^223^Ra	11.44 d	α	5979 (100%)	^223^Ra-dichloride (Xofigo^®^) * for bone metastasis	[90]
^166^Ho	26.8 h	β^−^ γ	1774 (49.9%) 80.57 (6.6%)	^166^Ho-chitosan ***** for liver cancer	[52]

Note: * FDA approved. ** Clinical trial phase III. *** Clinical trial phase II. **** Clinical trial Phase I. ***** Pre-clinical Studies.

**Table 4:** Bifunctional Chelating Agents (BFCAs).

BFCA	Radionuclide Compatibility	HOMO–LUMO ^a^	LogK_ML_^b^	References
DOTA	^111^In, ^86/90^Y, ^44/47^Sc, ^212/213^Bi, ^68^Ga, and ^177^Lu.	NA	23.9 (^111^In); 24.4 (^86/90^Y); 27.0 (^44/47^Sc); 30.30 (^212/213^Bi); 21.3 (^68^Ga); 25.41 (^177^Lu)	[109,110,111,112,113]
TCMC	^203^Pb and ^212^Pb	NA	> 19	[109,114,115,116,117,118,119,120,121]
DOTATATE	^68^Ga, ^111^In, ^90^Y, and ^177^Lu	NA	23.9 (^111^In); 24.4 (^90^Y); 21.3 (^68^Ga); 25.41 (^177^Lu)	[109,110,122]
DOTATOC	^111^In and ^90^Y	NA	NA	[123]
DOTANOC	^177^Lu	NA	NA	[122,123,124,125]
DTPA	^111^In, ^90^Y, ^177^Lu, ^64^Cu, and ^68^Ga	NA	29.5 (^111^In); 25.5 (^68^Ga); 21.4 (^64^Cu); 22.6 (^177^Lu)	[105,106,109,126]
1B4M-DTPA	^111^In and ^90^Y	NA	NA	[127,128,129]
CHX-A”-DTPA	^111^In, ^90^Y, ^177^Lu, and ^212/213^Bi	NA	NA	[103,130,131,132,133]
NOTA	^68^Ga and ^64^Cu	NA	31.0 (^68^Ga); 21.6 (^64^Cu)	[109,134,135,136,137,138,139]
NODAGA	^64^Cu	NA	>19.9	[140,141]
NODASA	^68^Ga and ^111^In	NA	30.9	[142,143]
NETA	^177^Lu, ^90^Y, and ^205/206^Bi	NA	NA	[144,145,146]
TETA	^64^Cu	NA	21.9	[109,147,148,149,150]
CB-TE2A	^64^Cu	NA	NA	[105,109,151,152,153,154]
H_2_dedpa	^68^Ga	NA	28.1	[155,156,157,158,159]
H_4_octapa	^111^In and ^177^Lu	NA	28.8 (^111^In); 20.1 (^177^Lu)	[160,161]
H_2_CHXdedpa	^67^Ga and ^111^In	NA	28.11	[115,162]
H_4_CXHoctapa	^67^Ga and ^111^In	NA	27.16	[163,164]
HYNIC	^99m^Tc and ^186^Re	NA	NA	[165,166,167]
EDDA/HYNIC-TOC	^99m^Tc and ^111^In	NA	NA	[168]
Sar bicyclic chelators	^64^Cu	NA	NA	[169,170,171,172,173]
T3,4BCPP	^99m^Tc and ^188^Re	HOMO: −0.273 LUMO: −0.242 of ^99m^Tc, HOMO: −0.273 LUMO: −0.243 of ^188^Re	NA	[174,175,176,177,178]
N(NOEt)_2_ isomers	^99m^Tc	ΔHOMO–LUMO anti = 1.080 ΔHOMO–LUMO syn-exo = 1.240 ΔHOMO–LUMO syn-endo = 1.650	NA	[179,180,181]
HBED-CC	^99m^Tc and ^68^Ga	(^99^m-Tc) ΔLUMO–HOMO = 4.917	38.5	[109,182,183]
PCTA-NCS	^177^Lu	NA	NA	[184,185,186]
MANOTA	^64^Cu	NA	NA	[187]
THP	^68^Ga	NA	14.2	[188,189,190,191,192]
DFO, DFO*, and DFOcyclo*	^89^Zr	NA	NA	[193,194,195,196]

^a^ = the lower HOMO–LUMO energy gap, compound less stable. ^b^ = high thermodynamic constant (LogK_ML_ > 18), compound more stable.

**Table 5:** Pharmacokinetic-modifying Linkers.

PKM Linkers	Structure	References
EGS	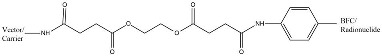	[197]
DSS	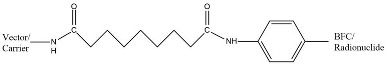	[197]
EMCS-Bz	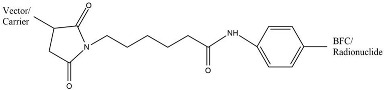	[198]
MESS-Bz	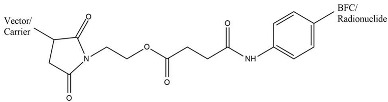	[198]
MIH	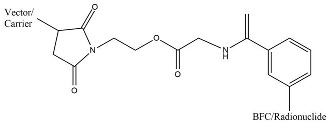	[198]
N4	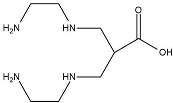	[199,200,201]
p-aminomethylaniline-diglycolic acid	NA	[202,203]
PEG	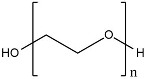	[203,204]

## Data Availability

Not applicable.
